# Bioactive Properties of Enzymatic Gelatin Hydrolysates Based on In Silico, In Vitro, and In Vivo Studies

**DOI:** 10.3390/molecules29184402

**Published:** 2024-09-16

**Authors:** Fenny Crista A. Panjaitan, Sin-Ting Shie, Sung Hoon Park, Tesalonika Sevi, Wen-Ling Ko, Rotimi E. Aluko, Yu-Wei Chang

**Affiliations:** 1 Marine Products Processing Study Program, Marine and Fisheries Polytechnic of Jembrana, Bali 82218, Indonesia; fennycap@gmail.com; 2 Department of Food Science, National Taiwan Ocean University, Keelung 20224, Taiwan; cindy70255@gmail.com (S.-T.S.); tesalonika.sevi@gmail.com (T.S.); sunnyko0414@gmail.com (W.-L.K.); 3 Department of Food and Nutrition, College of Life Sciences, Gangneug-Wonju National University, Gangneung 25457, Republic of Korea; sungpark@gwnu.ac.kr; 4 Department of Food and Human Nutritional Sciences, University of Manitoba, Winnipeg, MB R3T2N2, Canada

**Keywords:** ACE-I, acetylcholinesterase, bioactive peptides, MORRIS water maze, prolyl hydroxylase

## Abstract

This current study aims to analyze the potential bioactivities possessed by the enzymatic hydrolysates of commercial bovine, porcine, and tilapia gelatins using bioinformatics in combination with in vitro and in vivo studies. The hydrolysate with superior inhibition of angiotensin converting enzyme (ACE) activity was used to treat the D-galactose (DG)-induced amnesic mice. In silico digestion of the gelatins led to the identification of peptide sequences with potential antioxidant, ACE-inhibitory, and anti-amnestic properties. The results of in vitro digestion revealed that the <1 kDa peptide fraction of porcine gelatin hydrolysate obtained after 1 h digestion with papain (PP) (PP1, <1 kDa) potently inhibited ACE, acetylcholinesterase, and prolyl endopeptidase activities at 87.42%, 21.24%, and 48.07%, respectively. Administering the PP1 to DG-induced amnesic mice ameliorated the spatial cognitive impairment and Morris water maze learning abilities. The dentate area morphology in the PP1-treated mice was relatively similar to the control group. In addition, PP1 enhanced the antioxidant capacity in the DG-induced amnesic mice. This study suggests that PP1 could serve as a potential treatment tool against oxidative stress, hypertension, and neurodegenerative diseases.

## 1. Introduction

Gelatin is a biopolymer derived from animal skin, bone, or connective tissue collagen through heating, hydrolysis, and denaturation. Many studies have shown that gelatin can exhibit significant antioxidant activity, which helps eliminate excess oxidants [1,2,3]. Antioxidant activity is crucial for preventing oxidative stress caused by reactive oxygen species (ROS). The excessive ROS production or impaired antioxidant capacity contributes to cell or tissue damage, leading to chronic diseases [4]. The oxidative stress-induced oxidation of proteins, lipids, glycation, and DNA is linked to hypertension and Alzheimer’s disease. In addition, previous research has indicated that antioxidants can potentially reduce the incidence and symptoms of hypertension [5] and Alzheimer’s disease [6,7,8].

Hypertension, a significant risk factor causing cardiovascular diseases, is regulated by angiotensin-I converting enzyme (ACE-I) mechanisms. ACE converts angiotensin-I into angiotensin-II, which plays a role in vasoconstriction and increases blood pressure [9]. Numerous studies have demonstrated that gelatin hydrolysates from various sources can effectively manage hypertension to some extent [10,11,12]. Furthermore, hypertension is the leading factor in strokes, which are a common onset of cognitive impairment and the development of Alzheimer’s disease. Moreover, the ACE gene has also been studied to have a strong association with neurological diseases [13,14].

According to Nichols, et al. [15], the number of dementia cases globally, estimated at 57.4 million in 2019, is expected to rise to 152.8 million by 2050. The pathogenesis of neurodegenerative disorders is associated with the role of acetylcholinesterase (AChE) in terminating acetylcholine (ACh), which is the main neurotransmitter [16]. Therefore, AChE inhibitors are needed to regulate the AChE level to treat these diseases. Kim, et al. [17] reported that gelatin obtained from pig skin could exhibit anti-amnestic activity, preventing dementia by inhibiting AChE. Additionally, prolyl endopeptidase (PEP) activity is associated with neurological diseases such as Alzheimer’s, amnesia, and schizophrenia [18].

Several studies have shown that food peptides can inhibit hypertension and neurological diseases, and can also treat oxidative stress [19,20]. For instance, peptides from *Ziziphus jujuba* fruits have been found to act as DPPH inhibitors (IC_50_ = 0.75 mg/mL), AChE inhibitors (IC_50_ = 0.58 mg/mL), and also ACE inhibitors (IC_50_ = 0.060 mg/mL) [21,22]. Moreover, porcine skin gelatin hydrolyzed with prolyl endoproteinase showed potent peptides with an ACE IC_50_ of 51.11 µM [23], while peptides generated with Flavourzyme^®^ hydrolysis showed potentially strong antioxidant properties and improved cognitive function in mice [17]. Previous research works have extracted gelatin from various natural resources and analyzed their biological activities [11,23,24,25,26]. However, fewer studies have investigated the bioactivities of commercial gelatin produced by industries. Therefore, this study aims to hydrolyze commercial gelatins from porcine, bovine, and tilapia sources and investigate their bioactive peptides as antioxidant, antihypertensive, and anti-amnestic agents through in silico, in vitro, and in vivo analyses.

## 2. Results and Discussion

### 2.1. In Silico Analysis of Protein Sequences

**Figure 1:**
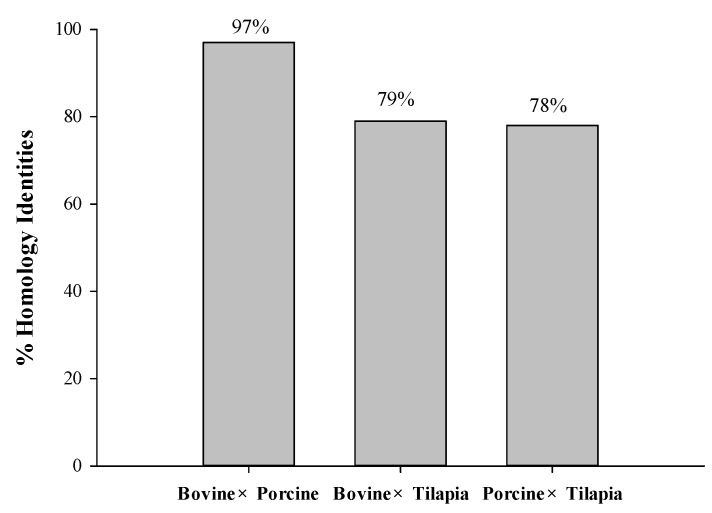
The homology identities of bovine, porcine, and tilapia collagen protein sequences.

A homology study was carried out using the Basic Local Alignment Search Tool (BLAST) analysis of protein sequences obtained from the UniProt KB database, such as bovine collagen alpha-1 (I) chain (P02453), Porcine alpha-1 chain of type I collagen (A0A1S7J210), and tilapia collagen type I alpha 1 (G9M6I5). The results revealed that sequence alignments of bovine, porcine, and tilapia collagen have a comparatively high identity value ranging from 78 to 97%, as depicted in Figure 1. Higher percentages of identity scores indicate better alignment and homology between the protein sequences [23]. The homology between their sequences might contribute to the similar potential bioactivities derived from these homologous proteins.

**Figure 2:**
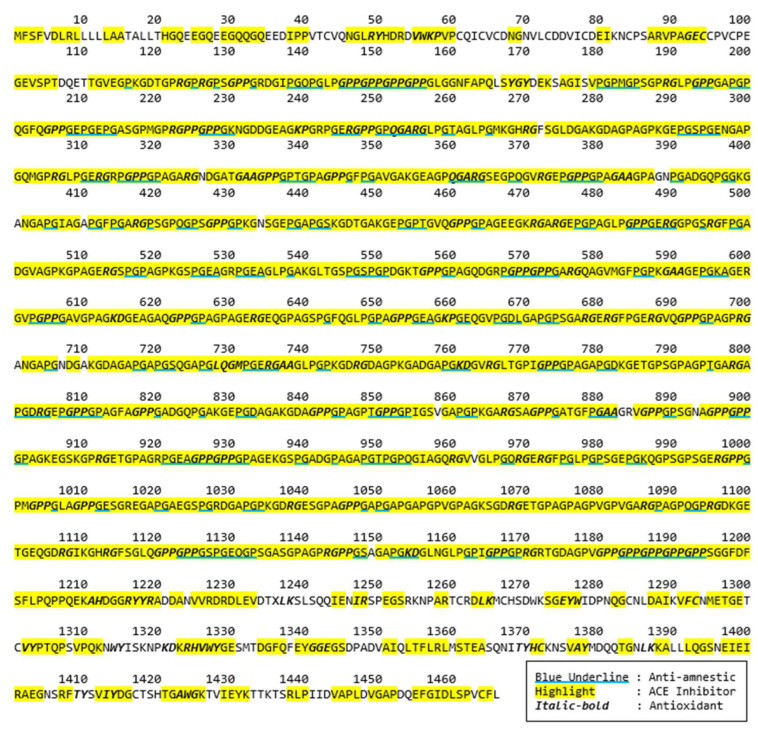
Potential bioactive peptides of Porcine alpha-1 chain of type I collagen (A0A1S7J210).

Bioactive peptides from these selected proteins were further identified. A bioactivity analysis in this study was initially conducted using computational approaches to predict the potential bioactive peptides released from the primary protein sequences. The BIOPEP-UWM database was used to predict potential bioactive peptides embedded in the selected protein sequences. The computational results of bioactive peptides profiling for the Porcine alpha-1 chain of type I collagen (A0A1S7J210) are illustrated in Figure 2. A number of bioactive peptides were identified based on their activities, such as anti-amnestic, angiotensin converting enzyme (ACE) inhibitors and antioxidative peptides. According to the data, most of these peptides are dipeptides with a few tripeptides. In addition, several overlapped peptides revealed different bioactivities, for example GE’s roles as an ACE inhibitor, while GEC is an antioxidative peptide.

Furthermore, a hydrolysis simulation was also carried out with bromelain and papain using the BIOPEP-UWM database. Lafarga and Hayes [27] demonstrated that a computer simulation can delineate the release of active peptides from sequences of specific proteins and screen enzymes that potentially produce bioactive peptides. Potential biological activities and the frequency (A_E_) of potential bioactive peptides generated within protein sequences after the hydrolysis simulation are listed in Table 1.

According to the results, ACE-inhibitory peptides were predominantly produced during the BIOPEP-UWM simulation, followed by anti-amnestic and antioxidative peptides. The A_E_ values of ACE inhibitors revealed by bromelain and papain ranged between 0.0919 and 0.0971, as well as 0.1091 and 0.1113, respectively. The A_E_ values of anti-amnestic peptides ranged from 0.0402 to 0.0428 for bromelain and from 0.0178 to 0.0207 for papain. In addition, antioxidant peptides showed the lowest frequencies at 0.0007 and 0.0014, based on simulated hydrolysis by bromelain and papain, respectively. The predictive results indicate that papain could hydrolyze proteins effectively to generate ACE inhibitors, while bromelain was expected to produce more antioxidant and anti-amnestic peptides. Bioinformatics studies have been successfully applied in predicting bioactivities and determining suitable proteases to release bioactive peptides from food proteins, such as tilapia skin [28] and giant grouper egg [29]. Nevertheless, an in vitro study should be conducted to confirm the theoretical results obtained from the computational test.

### 2.2. Proximate Composition of Gelatins

The proximate compositions of commercial bovine bone, porcine bone, and tilapia skin gelatins were measured and Table 2 shows that crude protein was the major component at 85.10 ± 0.82%, 83.56 ± 0.60%, and 83.31 ± 0.37%, respectively. The crude protein content of commercial tilapia skin gelatin was higher than the other studied commercial gelatins (*p* < 0.05), and was also relatively higher than the crude protein extracted from tilapia skin (80.75 ± 0.86%) obtained from other studies [28]. Alipal, et al. [30] reviewed that gelatin extracted from fish skin and scale contained protein levels ranging from 85 to 90%.

Furthermore, the moisture contents of gelatin from bovine, tilapia, and porcine sources were significantly different (*p* < 0.05), ranging from 13.29 to 14.63%. According to the proximate composition released by the Gelatin Manufactures Institute of America [31], the moisture content of dried gelatin should be <10%, which is relatively lower than that of the studied gelatins. Moreover, bovine gelatin revealed the highest percentage (*p* < 0.05) of ash content (0.42 ± 0.01%), followed by tilapia (0.21 ± 0.00%) and porcine (0.04 ± 0.01%). Sultana, et al. [32] reported that the ash content of gelatin ranges from 0.3 to 2%, which is affected by the filtration process through anion/cation exchange columns, resulting in the reduction in gelatin’s mineral or ash level.

### 2.3. Degree of Hydrolysis

**Figure 3:**
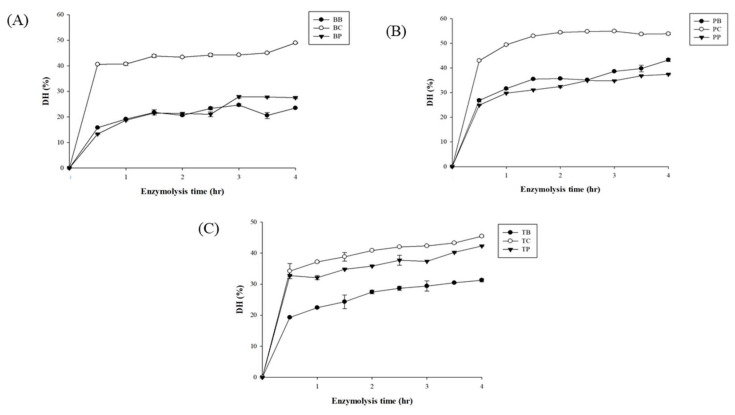
The degree of hydrolysis (DH) of bovine (**A**), porcine (**B**), and tilapia (**C**) gelatin hydrolysates hydrolyzed by bromelain (BB, PB, TB), collagenase (BC, PC, TC), and papain (BP, PP, TP) separately.

Gelatin proteins were hydrolyzed with proteases, such as bromelain, papain, and collagenase. The degree of hydrolysis (DH) from each sample was measured every thirty minutes, as shown in Figure 3. During the first half hour of digestion, the rates of collagenase-catalyzed bovine and porcine hydrolysis increased significantly compared to those of bromelain and papain, then they reached a plateau. In addition, the rate of hydrolysis in tilapia skin digested with collagenase and papain was almost at a similar level of DH and gradually increased.

The degree of hydrolysis, protein content, and yield were measured as listed in Table 3. Porcine, bovine, and tilapia hydrolyzed with collagenase (PC, BC, and TC) revealed high DH (*p* < 0.0.5) values at 53.89%, 48.95%, and 45.44%, respectively. A previous study conducted by Lee, Kim, Kim, Kim, Hwang, Lim, Moon, Jeon, Jeon and Ahn [2] also found that the DH of duck skin hydrolyzed by collagenase exhibited the highest percentage (48.70 ± 3.28%) when compared to alcalase, flavourzyme, neutrase, papain, pepsin, protamex, trypsin, and α-chymotrypsin. Collagenase effectively digests gelatin protein due to the capacity to bind and unwind the triple-helical protein structure before hydrolyzing it [33].

After 4 h of hydrolysis, PP had the highest protein content at 89.57 ± 0.75%, followed by TB at 87.54 ± 0.91% (*p* > 0.05). However, TB showed the highest percentage of yield (67.98 ± 15.67%), followed by PP (62.52 ± 2.85%) (*p* > 0.05). BB, on the other hand, produced the lowest percentage (*p* < 0.05) of protein content and yield at 69.49 ± 0.22% and 29.84 ± 0.93%, respectively. These results were in line with the DH of BB, which was also the lowest. The lower DH value for bovine hydrolysates may be due to gelatin’s structure, which is closely bound to the mineral phase, forming a complex interlocking structure [34].

### 2.4. Antioxidant Activities

Antioxidant activities were measured as shown in Table 3. The DPPH radical scavenging activity was used to evaluate the presence of radical scavenging antioxidants in natural sources [2]. The DPPH radical scavenging assay was conducted with a gelatin hydrolysate concentration of 0.45 mg/mL. Results showed that bovine groups (BB, BC, and BP) possessed a potent scavenging activity at 19.20 to 22.53% (*p* > 0.05). PC was noticeably higher among the porcine groups, at 18.05% (*p* < 0.05), than PB and PP. Moreover, the DPPH radical scavenging activity observed in the tilapia groups showed that TP had the highest capacity at 15.52%, followed by TC and TB. A previous study by Shiao, et al. [35] reported that tilapia gelatin hydrolyzed by pepsin could exhibit a strong DPPH scavenging activity at 84%. Overall, bovine hydrolysates were the most effective in scavenging DPPH radicals, followed by porcine and tilapia hydrolysates. According to these findings, raw materials influenced the capacity to scavenge the DPPH radicals, which may be due to the variations in the type of amino acid residues present in the hydrolysates. Chi, et al. [36] confirmed that the composition of amino acid sequences, such as hydrophobic and aromatic amino acid residues, affected the performance of DPPH radical scavenging abilities.

In FRAP analysis (1.5 mg/mL), BC and BP demonstrated more potent abilities, to reduce Fe^3+^ to Fe^2+^ (≥0.008 mmol Fe^2+^/mg protein), than BB. Manosroi, et al. [37] stated that papain exhibited a higher scavenging activity than bromelain. PB and PP also showed similar trends and were higher than PC. In addition, tilapia hydrolysates (TB, TC, and TP) generated a low range of activities in reducing the ferric iron, at 0.004–0.007 Fe^2+^/mg protein. Differences between the hydrolysates could be influenced by the electron-donating ability of active peptides, known as the stoichiometric factor [38]. Additionally, the DH value observed in each hydrolysate did not give any notable influence on Fe-reducing power. Similar findings have also shown that the alcalase hydrolysates of squid tunic gelatin showed an insignificant Fe^3+^-reducing ability as the DH increased [39].

Collagenase produced effective gelatin hydrolysates with a good metal ion chelating effect ranging from 46.66 to 69.75%, which was better than bromelain hydrolysates (13.24–56.25%) and papain hydrolysates (24.02–34.41%) at 1.5 mg/mL. The results showed that collagenase generated a high degree of hydrolysis in the gelatin substrate, correlating to significant hydrolysis activity. Protein hydrolysates obtained from high DH values were composed of low molecular weight peptides, which are linked to the potent antioxidant activity [40].

The results of superoxide anion scavenging capacity showed that BC and PC had the highest scavenging activity at above 30%, while TC was at 29.01% (*p* < 0.05). In the bromelain group, the highest superoxide anion scavenging activity was produced by porcine at 16.03%. In addition, there was no considerable difference in the superoxide anion scavenging capacity (*p* > 0.05) between porcine, bovine, and tilapia hydrolysates treated with papain.

### 2.5. ACE-I Inhibitory Activity

The ACE-I inhibitory activity was measured at a concentration of 1 or 2 mg/mL gelatin hydrolysates, as shown in Table 3. Collagenase hydrolysis produced peptides with better ACE-I inhibitory activities, ranging from 40 to 50%. The results are in line with the high DH of collagenase digestion from bovine, porcine, and tilapia proteins at 48.95%, 53.89%, and 45.44%, respectively. According to the results, PP (2 mg/mL) possessed the highest ACE-I inhibitory activity among other hydrolysates at 60.94%. Therefore, it was chosen for further bioactivity assay, including AChE and PEP inhibitory analyses.

A similar finding was obtained from ribbon fish hydrolysates, revealing that a high DH produced a higher ACE-I inhibitory activity due to smaller peptides distribution [41]. Nevertheless, porcine gelatin digested with papain (PP) generated the highest percentage of ACE inhibition among the hydrolysates at 60.94% (*p* < 0.05). Additionally, the hydrolysate concentration influenced the capacity to inhibit ACE-I. PP hydrolysates at 1 mg/mL (94.76%), which exhibited a better activity than those at 2 mg/mL (60.94%). Wu, et al. [42] also reported that increasing the substrate concentration gave reverse activities in inhibiting ACE-I.

### 2.6. Effect of Hydrolysis Time

PP hydrolysates possessing a potent ACE-I inhibitory activity were further analyzed at 1-hour (PP1) and 4-hour (PP4) hydrolysis periods. PP1 and PP4 were then characterized and presented in Table 3. From the results, the DH of PP1 was significantly lower than that of PP4 (*p* < 0.05), which was influenced by the shortened period of hydrolysis. Noman, et al. [43] reported that prolonging the incubation time will result in an increment in DH due to the extensive action of the enzyme to hydrolyze substrate. Yathisha, Vaidya and Sheshappa [41] reported that a high DH is dominated by a low molecular weight peptide resulting in high solubility. Conversely, the protein content and yield of PP1 showed a high percentage at 94.48% (*p* < 0.05) and 65.23% (*p* > 0.05), respectively. Besides DH, amino acid profiles are vital in exhibiting the ACE-I inhibitory activity [44]. The presence of amino acids composed in PP1 hydrolysates are presented in Table 4.

The antioxidant activities were measured at a 1 mg/mL hydrolysate concentration. Results revealed that PP1 (8.53%) and PP4 (8.08%) were far lower in the DPPH scavenging activity than the glutathione (GSH) (50.90%) (Figure S1). PP1 and PP4 could chelate metal ion at 11.83% and 16.74%, respectively. At a higher hydrolysates concentration (3 mg/mL), PP1 and PP4 chelated 40.70% and 45.21% of the metal ions, respectively. Furthermore, in the FRAP test, PP1 and PP4 inhibited at 0.20 and 0.35 mmol Fe^2+^/mg protein, respectively. However, they were lower than GSH (0.05 mg/mL) at 0.59%. In the superoxide radical scavenging test, the PP1 (18.53%) and PP4 (17.69%) activity were far below GSH control (71.86%). According to the antioxidant results, the hydrolysis time did not effectively improve antioxidant activities. A similar finding was reported that there were no significant improvements in antioxidant activities by extending the hydrolysis period [10].

PP1 (96.56%) and PP4 (94.76%) exhibited a potent inhibition of ACE activity at 1 mg/mL. Corresponding to these findings, 1-hour and 4-hour hydrolysis did not give significant differences in ACE-I inhibition (*p* > 0.05). Therefore, PP1 was chosen to be further fractionated to observe the activities of antioxidant, ACE-I, AChE, and PEP inhibition due to shorter hydrolysates preparation, which favors cost and time reductions.

### 2.7. Peptides Fractionation

Protein fractionation was conducted to assess the capacity of peptides distributed in three fractions (<1 kDa, 1–5 kDa, and 5–10 kDa). Table 5 showed that lower molecular weight (<1 kDa) peptides possessed better ACE-I, AChE, and PEP inhibition activities. Generally, the antioxidant capacity did not improve after the ultrafiltration process. Presumably, peptides with antioxidant activity were reduced and separated due to fractionation [45]. Moreover, the <1 kDa peptides (1 mg/mL) effectively inhibited ACE at 87.42% (*p* < 0.05). The result is consistent with a previous study by UG, Bhat, Karunasagar and BS [44], which stated that fractions <1 kDa showed a higher ACE-inhibitory activity compared to 1–5 kDa, 5–10 kDa, and > 10 kDa. Lin, Alashi, Aluko, Sun Pan and Chang [24] reported that the <1 kDa fraction from pepsin-hydrolyzed tilapia frame hydrolysates reduced blood pressure in spontaneously hypertensive rats.

Anti-amnestic properties were also studied. Similar results were obtained for AChE and PEP inhibition, showing that the <1 kDa peptides (10 mg/mL) had the highest AChE inhibitory capacity at 21.24% (*p* < 0.05). Moreover, PEP (1 mg/mL) was strongly inhibited at 48.07%. PEP inhibition by the 1–5 kDa and 5–10 kDa peptide fractions was not detected, even when the concentration increased to 10 mg/mL. According to these findings, the MW distribution was crucial in possessing PEP inhibitory peptides. Wang, et al. [46] reported that the papain hydrolysates of porcine gelatin exhibited good neuroprotective activity with peptides <3 kDa. High levels of PEP are associated with neuropathological disorders such as depression, dementia, and Alzheimer’s disease [47]. PEP cleaves neuro-active peptides with a specific cleavage at the C-side of proline, which plays vital roles in the brain [48]. Therefore, PEP-inhibitory peptides have relevant roles as agents that could maintain normal neuronal functions.

### 2.8. Animal Behavior Test

As previously reported by Canavan and O’Donnell [5], hypertension is a risk factor for cognitive impairment and dementia. Therefore, a further analysis was conducted to observe the effect of PP1. D-galactose (DG)-induced mice were used in this study to observe the role of gelatin peptides in treating performance loss and learning degradation.

#### 2.8.1. Physiological Observation of Treated Mice

The body weight, daily food and water intake, and weights of mice’s relative brain, liver, and fat tissue are shown in Table 6. The results indicated that there was no significant influence of DG on the physical condition of mice after 10 weeks (*p* > 0.05) compared to the control (CON). DG-fed mice have been studied to exhibit incremental cognitive and movement performance loss. These symptoms mimic those of aging, making it as an accelerated aging model for anti-amnestic testing [49]. Zhen, et al. [50] and Zhao, et al. [51] reported that the DG injection in mice (at doses of 100 and 1000 mg/kg/day) did not affect the physiological parameters after 8 weeks of administration.

#### 2.8.2. Morris Water Maze (MWM) Test for In Vivo Analysis

**Figure 4:**
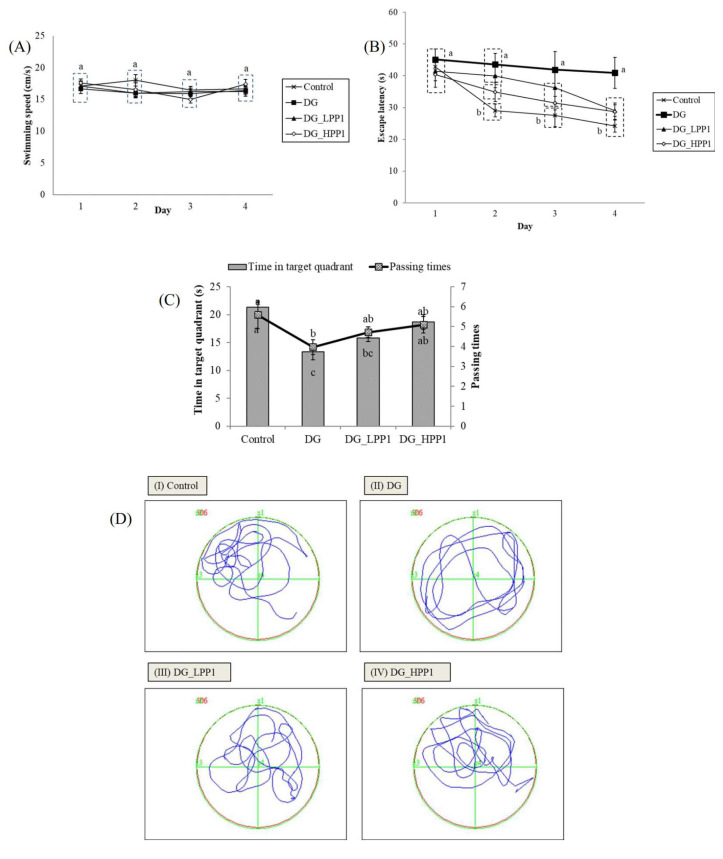
Learning and memory abilities of experimental mice with (**A**) swimming speeds, (**B**) escape latency, (**C**) periods and passing times spent in the target quadrant (zone II), and (**D**) the swimming pathway of experimental mice groups (I–IV). Data are given as mean ± standard deviation (*n* = 6). Different letters (a,b,c) indicate significant differences (*p* < 0.05).

In the reference memory test depicted in Figure 4A, mice underwent four trials per day for four consecutive days. The results showed that the DG group exhibited a consistent plateau in escape latency over the four days, significantly longer (*p* < 0.05) on Day 4 at 40.90 s. The administration of PP1 markedly reduced escape latencies, substantially decreasing them on Day 4 (DG_LPP1: 29.06 s and DG_HPP1: 28.72 s). Figure 4B displays swimming performances ranging from 15.00 to 18.00 cm/s. Neither DG nor PP1 induction influenced the mice’s swimming performance compared to the CON group (*p* > 0.05).

Figure 4C illustrates that the DG group spent the shortest time (13.35 s) in the target quadrant (Zone II) during the probe test. Increasing concentrations of PP1 prolonged the time spent by mice in the target quadrant (DG_LPP1: 15.78 s and DG_HPP1: 18.66 s). Additionally, DG_HPP1 spent an amount of time that was insignificantly different (*p* > 0.05) compared to the CON group. Corresponding findings were also noted in the frequency of crossings over the target quadrant (Zone II). DG had the lowest frequency of crossings in the target zone, whereas the administration of PP1 tended to increase the number of crossing over the target quadrant to around four to five times. These results indicate that mice treated with PP1 exhibited enhanced ability to remember the target quadrant where the platform was located during training. PP1 mitigated cognitive impairment caused by DG in mice. Wang, et al. [52] reported that mice supplemented with oyster protein hydrolysates had better spatial memory and learning abilities in both an MWM and a dark/light avoidance test. Another study involving the administration of porcine brain hydrolysates into Aβ (1–40)-infused rats also significantly improved the spatial performance, as well as the performance in reference and working memory tests in water maze tasks [53].

Figure 4D illustrates the swimming pathways. The blue line indicated the swimming pathways of mice, while the green line illustrated the border of each quadrant. The CON group mainly swam in Zone II, indicating focused and directed swimming. In contrast, the DG group exhibited directionless and disordered paths, swimming around the pond without a clear strategy. The DG_LPP1 group swam around the center of the pond, focusing on in quadrants I and II. The DG_HPP1 group showed focused swimming around the target zone, indicating that the mice were actively seeking the platform. These results indicate that administering PP1 to DG-induced mice improved their memory and learning performances, as evidenced by more purposeful swimming behaviors towards the target zone.

#### 2.8.3. Antioxidant Capacities on Mice Brain

The antioxidant capacities of the mice brain tissue were assessed to observe the effect of PP1 in reducing oxidative stress associated with memory impairment (Table 6). The results indicated that the DG group exhibited the lowest levels of TBARS (Thiobarbituric Acid-Reactive Substances) and TEAC (Trolox Equivalent Antioxidant Capacity). Moreover, the administration of PP1 increased both TBARS and TEAC values compared to the DG group.

SOD (Superoxide Dismutase) and GPx (Glutathione Peroxidase) activities were also measured to assess oxidative stress in mice brain cells. Both activities were significantly reduced (*p* < 0.05) in the DG group compared to the CON group. However, in the DG_HPP1 group, the SOD activity was notably improved at 1.88 ± 0.15 unit/mg protein (*p* < 0.05). Furthermore, PPI administered to DG-induced mice enhanced the GPx activity. The increased SOD and GPx activities observed in DG-induced mice treated with PP1 suggest potential therapeutic benefits in conditions related to memory impairment, as supported by the MWM study’s results.

#### 2.8.4. Histopathology of Mice Brain Tissue

**Figure 5:**
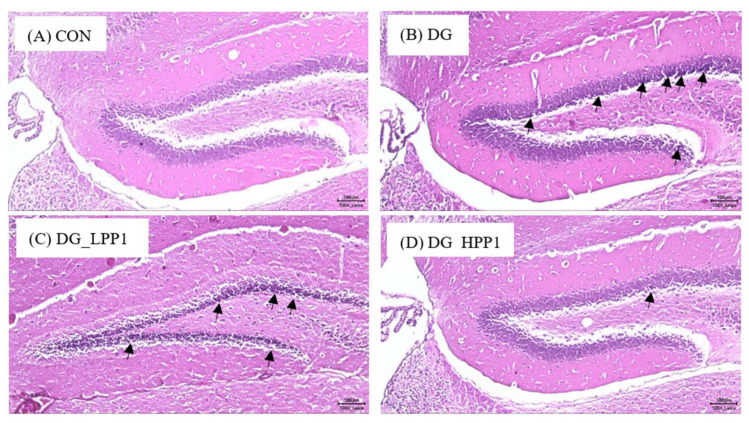
Morphological features of the dentate gyrus area from (**A**) CON, (**B**) DG, (**C**) DG_LPP1, and (**D**) DG_HPP1 groups. The presence of dark neurons characterized by contracted neuron bodies (black arrows) (magnification: 100×). Scale bar: 100 μm.

The hippocampus, situated beneath the cerebral cortex, plays an crucial role in memory function, with the dentate gyrus particularly involved in spatial memory discrimination [54]. Therefore, the morphology of the dentate gyrus area in the mouse hippocampus was observed using hematoxylin and eosin staining, as shown in Figure 5. Neurons throughout the granule cell layer (GCL) and the subgranular zone (SGZ), which borders the hilus and GCL, were completely stained with hematoxylin. Comparing with the CON group, the DG group exhibited a higher prevalence of shrunken neurons stained dark purple, indicated by black arrows, suggesting many neurons with condensed cytoplasm. However, as the dosage of PP1 increased, the number of dark neurons in the dentate gyrus area noticeably decreased. Furthermore, the morphological features of the dentate gyrus in the DG_HPP1 group closely resembled those of the CON group. These findings correspond with the results of the water maze test, indicating that DG_HPP1 performed well on spatial memory tasks.

## 3. Materials and Methods

### 3.1. Materials

Commercial bovine bone and tilapia skin gelatins were provided by Jellice Co. Ltd., Pingtung, Taiwan, while commercial porcine skin gelatin was supplied by Gemfont Co. Ltd., Taipei, Taiwan. Bromelain (EC 3.4.22.32), collagenase (EC 3.4.24.3), and papain (EC 3.4.22.2), ACE from lung rabbit (≥2 units/mg protein), the substrate *N*-[3-(2-Furyl)acryloyl]-l-phenylalanyl-glycyl-glycine (FAPGG), AChE type VI-S from electric eel (200–1000 units/mg protein), prolyl endopeptidase (PEP) from *Flavobacterium* sp. (≥5.0 units/mg solid), acetylcholine iodide, Z-glycyl-L-proline-4-nitroanilide (Z-Gly-Pro-pNA), and Ellman reagent 5,5′-Dithiobis (2-nitrobenzoic acid) were all purchased from Sigma-Aldrich Co. (St. Louis, MO, USA). Other analytical-grade chemicals were also employed in this study.

### 3.2. In Silico Analysis

#### 3.2.1. Homology Study of Bovine, Porcine, and Tilapia Gelatin Sequences

Gelatin sequences obtained from UniProt (available at http://www.uniprot.org/; accessed on 20 May 2023), such as bovine collagen alpha-1 (I) chain (P02453), Porcine alpha-1 chain of type I collagen (A0A1S7J210), and tilapia collagen type I alpha 1 (G9M6I5) were performed for the homology study using BLAST (https://blast.ncbi.nlm.nih.gov/Blast.cgi; accessed on 20 May 2023). The percentage of identity was determined between those proteins.

#### 3.2.2. Bioactive Peptides Analysis by BIOPEP-UWM Database Tools

Bioactivities were predicted using the BIOPEP-UWM database (available at https://biochemia.uwm.edu.pl/biopep/start_biopep.php; accessed on 20 May 2023). The enzymatic hydrolysis was simulated using bromelain and papain via the BIOPEP-UWM “enzyme action” tool. The frequency of bioactive peptides (AE) was calculated as AE = a/N, where a represents the number of bioactive peptides and N represents the total number of amino acid residues in the intact protein sequences.

### 3.3. Proximate Analysis

The moisture, ash, crude protein, and crude fat content of gelatins (bovine, tilapia, and porcine) were determined according to methods adopted from the Association of Official Analytical Chemists [55].

### 3.4. Preparation of Gelatin Hydrolysates

An enzymatic hydrolysis of commercial gelatin was conducted with some modifications based on the procedure by Noman, Xu, AL-Bukhaiti, Abed, Ali, Ramadhan and Xia [43]. Commercial gelatin was dissolved in deionized water at a ratio of 1:100 (*w*/*v*) solid-to-liquid and adjusted to the optimal conditions for each enzyme (bromelain: 50 °C, pH 7; collagenase: 37 °C, pH 7; papain: 55 °C, pH 7). Hydrolysis was performed by adding 1% (enzyme/substrate, *w*/*w*) of each enzyme. After 4 h of hydrolysis, the mixtures were placed in a water bath at 95 °C for 15 min to inactivate the enzymatic reactions and then cooled to ambient temperature. The hydrolysates, referred to as bovine–bromelain (BB), bovine–collagenase (BC), bovine–papain (BP), porcine–bromelain (PB), porcine–collagenase (PC), porcine–papain (PP), tilapia–bromelain (TB), tilapia–collagenase (TC), and tilapia–papain (TP), were lyophilized and stored at −20 °C until use. The protein content of the gelatin hydrolysates was determined using the Lowry method [56].

### 3.5. Degree of Hydrolysis (DH)

The degree of hydrolysis (DH) was determined using the *o*-phthalaldehyde (OPA) method as described by Charoenphun, et al. [57]. The OPA solution was freshly prepared with 100 mM sodium tetraborate 12.5 mL, 1.25 mL of 20% SDS, 20 mg of OPA in 0.5 mL methanol, 0.05 mL of 2-mercaptoethanol, and 10.7 mL of double-distilled water (ddH_2_O). The sample (10 µL), Gly-Gly-Gly standard (5 µL), and OPA solution (200 µL) were mixed and incubated for 100 s at 37 °C. Gelatin hydrolysates were then added to 6 N HCl and stirred for 24 h at 100 °C for a total acid analysis. Absorbance was measured at 340 nm using multiple readers (Multiskan Go, Thermo Fisher Scientific, Waltham, MA, USA). The *DH* (%) was calculated using the following equation:

(1) $$DH(\%)=\left[\frac{({NH}_{2}{)}_{tx}-({NH}_{2}{)}_{t0}}{({NH}_{2}{)}_{total}-({NH}_{2}{)}_{t0}}\right]\times 100\%$$

where (*NH*_2_)*_tx_* is the free amino groups at X min (mg/mL), and (*NH*_2_)*_total_* is the total amino groups by total acid hydrolysis (mg/mL). (*NH*_2_)*_t_*_0_ is the free amino groups at 0 min of hydrolysis (mg/mL).

### 3.6. Peptide Fractionation

The sample (10 mg/mL) was furtherly isolated with an ultrafiltration process using an Amicon stirred ultrafiltration unit (Millipore Corporation, Bedford, MA, USA) with 1, 5, and 10 kDa molecular weight cut-off (MWCO) membranes. Protein isolates were collected at certain molecular weights (<1 kDa, 1–5 kDa, and 5–10 kDa). Isolates were then lyophilized and stored at −20 °C until use.

### 3.7. Antioxidant Analysis

#### 3.7.1. DPPH Radical Scavenging Activity Assay

The DPPH• (2,2-diphenyl-1-picrylhydrazyl) radical scavenging activity was analyzed using a modified method from Girgih Abraham, et al. [58]. The sample was dispersed in 0.1 M sodium phosphate buffer (pH 7.0) containing 1% (*v*/*v*) Triton X-100. Hydrolysates (100 μL) were mixed with a 100 μL methanolic solution of 100 μM DPPH in a 96-well plate. The mixture was allowed to stand for 30 min in the dark, and the absorbance was read at 517 nm. The negative control groups used sodium phosphate buffer solution instead of the sample, and the positive control used glutathione (GSH). The DPPH radical scavenging activity was calculated as follows:

(2) $$DPPH•scavenging\;activity(\%)=\left(1-\frac{A_{sample}}{A_{control}}\right)\times 100\%$$

#### 3.7.2. Metal Ion Chelating Activity Assay

With some modifications, the metal ion chelating activity was determined based on the method by Xie, et al. [59]. Hydrolysates or GSH (500 μL) were mixed with 2 mM FeCl_2_ (25 μL) and deionized water (1.85 mL), followed by the addition of ferrozine solution (50 μL, 5 mM). The mixture was allowed to stand at ambient temperature for 10 min. A 200 μL aliquot was then transferred to a 96-well plate, and the absorbance was measured at 562 nm. The control group consisted of deionized water. The following formula was used to calculate the metal ion chelating activity assay:

(3) $$Metal\;chelating\;activity(\%)=\left(1-\frac{A_{sample}}{A_{control}}\right)\times 100\%$$

#### 3.7.3. Ferric-Reducing Antioxidant Power (FRAP) Assay

With some modifications, the ferric-reducing antioxidant power (FRAP) was measured using the procedure of Benzie and Strain [60]. The FRAP reagent was prepared by mixing 0.3 M acetate buffer, 10 mM TPTZ (in 40 mM HCl, pH 4.6), and 20 mM FeCl_3_ (pH 3.6) in deionized water at a 5:1:1 (*v*/*v*/*v*) ratio. The hydrolysate was dispersed in distilled water to achieve a final assay concentration of 1 mg/mL. The sample (40 μL) was shifted to a 96-well plate and mixed with the FRAP reagent (200 μL) at 37 °C. A solution of FeSO_4_·7H_2_O (0.025–0.15 mM) was used to create the standard curve. The absorbance, specified as Fe^2+^ nM per mg peptide, was measured at 593 nm.

#### 3.7.4. Superoxide Radical Scavenging Activity (SRSA) Assay

The superoxide radical scavenging assay was conducted based on the method by Siswoyo, et al. [61]. The sample (80 μL) was mixed with 50 mM Tris-HCl buffer (pH 8.3) containing 1 mM EDTA (40 μL) in a 96-well plate, followed by the addition of 1.5 mM pyrogallol in 10 mM HCl (40 μL). The absorbance was measured at 420 nm every minute (ΔAs) for 4 min. The control group (ΔAc) used distilled water. The following equation was applied to the enumerate scavenging activity:

(4) $$Superoxide\;radical\;scavenging\;activity\;\left(\%\right)=\left[1-{∆A}_{s}\min^{-1}/{∆A}_{c}\min^{-1}\right]\times 100\left(\%\right)$$

### 3.8. Angiotensin-I Converting Enzyme (ACE-I) Inhibition Assay

The ACE-I inhibitory activity was determined using the synthetic substrate *N*-[3-(2-furyl)acryloyl]-l-phenylalanyl-glycyl-glycine (FAPGG) based on the method by Girgih, et al. [62]: FAPGG (0.5 mM). The samples were mixed in a 50 mM Tris-HCl buffer containing 0.3 M NaCl, adjusted to pH 7.5. A 170 µL aliquot of 0.5 mM FAPGG was mixed with 10 µL ACE (0.5 U/mL, 25 mU final activity) and a 20 µL sample. The absorbance reduction at 345 nm was recorded every 3 min for 30 min at 37 °C using a Synergy H4 microplate reader (Biotek Instruments, Winooski, VT, USA). The Tris-HCl buffer was used as a control. The ACE-I activity was defined as the rate of reaction (ΔA/min) and calculated as follows:

ACE-I inhibition (%) = [1 − ΔAmin^−1^(sample)/ΔAmin^−1^(control)] × 100% (5)

where ΔAmin^−1^_(sample)_ is the ACE-I activity in the presence of peptides, and ΔAmin^−1^_(control)_ is the ACE-I activity in the absence of the peptides.

### 3.9. Anti-Amnestic Activity

#### 3.9.1. Prolyl Endopeptidase (PEP) Inhibition Assay

The PEP-inhibiting activity was implemented using the procedure of Sila, et al. [47]. An aliquot of 10 µL phosphate buffer (0.1 M, pH 7.0), 200 µL sample (1 mg/mL), and 20 µL of 2% Z-Gly-Pro-pNA (in 50% 1,4-dioxane) was mixed and incubated (37 °C, 10 min). The reaction was initiated by adding 20 µL of PEP (0.1 unit/mL). The absorbance of the sample (A_sample_) was observed by the release of p-nitroaniline at 410 nm for 30 min in a multiple reader. Phosphate buffer was the negative control (A_control_) and Z-Pro-proline (1 mg/mL) was a positive control. The following equation enumerated the inhibition activity:

(6) $$PEP\;inhibitor\left(\%\right)=\left[1-{∆A}_{\mathrm{sample}}{min}^{-1}/{∆A}_{\mathrm{control}}{min}^{-1}\right]\times 100\left(\%\right)$$

#### 3.9.2. Acetylcholinesterase (AChE) Inhibition Assay

The AChE inhibitory assay followed the method of Malomo and Aluko [63]. Gelatin hydrolysates (20 µL) were added to 130 µL sodium phosphate buffer (0.1 M, pH 7.5) before adding 3 mM DTNB (20 μL) and 15 mM acetylthiocholine (10 µL). The reaction was initiated by adding 20 μL AChE (0.5 U/mL final assay concentration) and incubated (15 min) at ambient temperature. A multiple reader was employed to measure the absorbance at 412 nm. The negative control was 0.1 M sodium phosphate buffer (pH 7.5), while the positive control was galantamine hydrobromide in buffer at 1 mg/mL. The activity was enumerated as follows:

(7) $$AChE\;inhibitor\left(\%\right)=\left(1-A_{\mathrm{sample}}/A_{\mathrm{control}}\right)\times 100\left(\%\right)$$

### 3.10. Animal Behavior Assessment

#### 3.10.1. Animals

The study was performed on thirty-two 7-week-old male ICR mice from the National Taiwan University College of Medicine (Taiwan) animal center. Before the experiment, mice were enabled to access food and water freely. They were put in a regulated cage with a temperature and humidity of 22 ± 2 °C and 60–80% in a 12 h light/dark cycle. After adapting for 1 week, the mice were separated randomly into a D-galactose (DG) group, D-galactose with a low concentration of hydrolysates (DG_LPP1) group, D-galactose with a high concentration of hydrolysates (DG_HPP1) group, and control (CON) group (Table 7) and treated for eight weeks. Food, water consumption, and the body mass of mice were documented every week. Animal testing was permitted by the Institutional Animal Care and Use Committee of National Taiwan University (IACUC No. NTU105-EL-00163). After the Morris water maze (MWM) test, mice fasted for eight hours before they were euthanized. Brain, liver, epididymal fat, and perirenal fat from the mice were removed and then weighed individually.

#### 3.10.2. Morris Water Maze (MWM) Test Preparation

**Figure 6:**
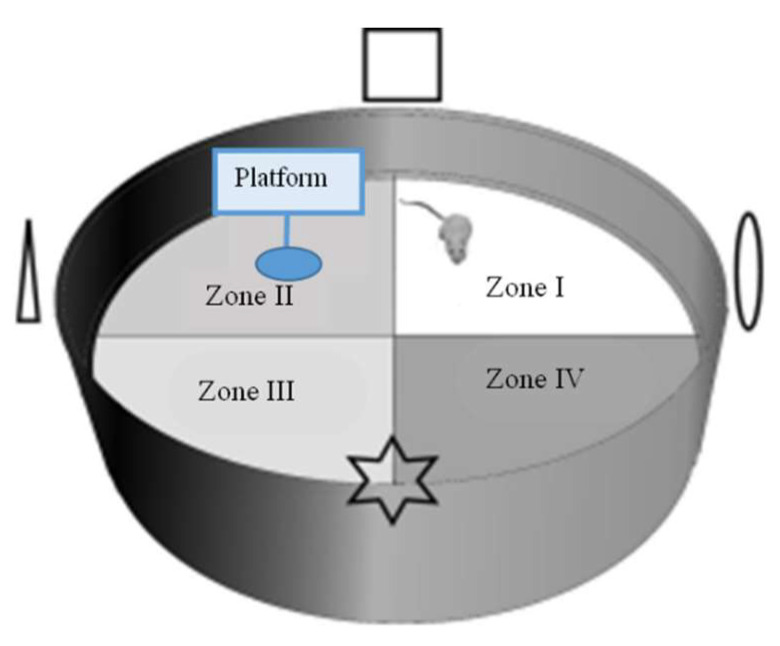
Morris water maze (MWM) test apparatus (adapted from Gonzalez-Perez, et al. [65]).

The MWM test was utilized based on Lu, et al. [64]. The MWM test was completed for six consecutive days in the eighth week of the experiment in an apparatus (Figure 6) consisting of a round pool (diameter: 100 cm, height: 80 cm), a platform (diameter: 4.3 cm, height: 16 cm), and containing water (23 ± 1 °C), which was added by skim milk powder and the food coloring agent (Blue, Ever Style Foodstuff Industrial Co., Ltd., Taipei City, Taiwan) to render it opaque. The pool was bounded into four quadrants (Zone I–Zone IV) with contrast spatial cues (square, triangle, circle, and star) placed on the pool’s interior, above the water’s surface. On day 1, red tape was located on the platform’s top (1–1.5 cm above the water surface) in the center to increase the platform’s visibility. In the memory test (days 2–5), each group received four types of test per day to discover the hidden platform. On day 6, the probe test was implemented. The platform was detached from the pool, and mice could swim for 60 s. The elapsed time by mice swimming in and the frequent exact crossing over the target zone (location of the platform before detachment) were noted in every trial. The swimming path was documented by a video recorder set above the pool and traced in the software of animal behavior (Singa Trace mouse II, Diagnostic and Research Instruments Co., Ltd., Taipei, Taiwan).

### 3.11. Brain Tissue Collection and Homogenates Preparation

**Figure 7:**
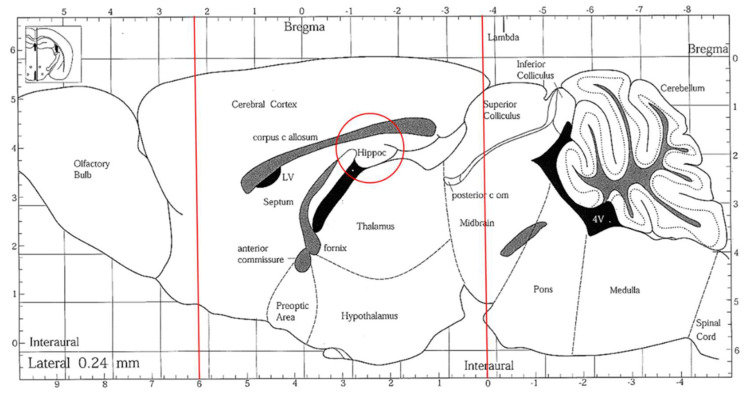
A sagittal section of the mouse brain. The brain tissues were sectioned, indicated by the red lines, and the hippocampus areas (red circle) were collected.

The preparation of brain tissue and homogenates was immediately conducted after mice were euthanized. Brain tissue was sectioned into several parts (Figure 7). For a histopathological assessment, the sections of brain tissues were located in a 10% formaldehyde solution (Merck Millipore Co., Darmstadt, Hesse, Germany). The rest of the brain tissue was set to a homogenous mixture on ice with the 9-fold volume of PBS (pH 7.4, including 0.25 M sucrose) using a homogenizer (Polytron, PT-2100, Kinematica AG, Lucerne, Switzerland) and then centrifuged (1000× *g*, 4 °C, 15 min). The supernatant was taken and stored at −80 °C for analysis. A Bio-Rad protein assay kit (catalog #500-0006; Bio-Rad Laboratories, Inc., Hercules, CA, USA) was used to quantify the supernatant protein concentration with the BSA standard.

### 3.12. Thiobarbituric Acid Reactive Substances (TBARS) Assay

The TBARS assay was measured based on the approach by Chan, et al. [66]. A total of 30 µL brain homogenates aliquot (10%) was added with a 45 µL TBA solution (J. T. Baker; Mallinckrodt Baker, Inc., Philipsburg, NJ, USA) and a 255 µL trichloroacetic acid-hydroxyl chloride (TCA-HCl) reagent (Sigma-Aldrich, St. Louis, MO, USA). Then, the mixture was subjected to heat (95 °C, 30 min), chilled on ice (10 min), and centrifuged (1000× *g* at 4 °C, 5 min). A total of 200 µL aliquot was measured at 532 nm using malondialdehyde extinction coefficients to be 1.56 × 10^5^ M^−1^ cm^−1^ by a microplate reader (Synergy H1 Hybrid Multi-Mode Microplate Reader, BioTek Instruments Inc., Winooski, VT, USA). PBS was the control group. The TBARS value was determined as follows:

(8) $$TBARS\left(nmol\;MDA\;eq./mg\;protein\right)=A_{s}\times 705.15/protein\;concentration\;(mg/mL)$$

### 3.13. Trolox Equivalent Antioxidant Capacity (TEAC) Assay

TEAC was assessed by Hung, et al. [67]. The regent was prepared by mixing 5 mL of ABTS (100 μM; Sigma-Aldrich, St. Louis, MO, USA), H_2_O_2_ (50 μM; Sigma-Aldrich, St. Louis, MO, USA), catalase (4.4 U/mL; Sigma-Aldrich, St. Louis, MO, USA), respectively, as well as 30 mL ddH_2_O. The reagent was vigorously mixed and reacted for 1 h in the darkness at ambient temperature. The TEAC value was determined by diluting 25 µL of brain homogenate in 10-fold PBS and reacting with 250 µL reagent in the dark for 10 min. The Trolox was used as a standard curve to calculate the TEAC value (nmol/mg protein) in the sample at 734 nm absorbance.

### 3.14. Superoxide Dismutase (SOD) Assay

The assessment of SOD in brain tissue was completed as referred to by Mueller, et al. [68]. The SOD value was measured by inhibiting SOD on pyrogallol autoxidation at 420 nm for 1 min. Brain homogenate (20 µL) was mixed with a 5-fold volume of PBS. The max. speed of SOD inhibition was set to the reaction of SOD standard (Sigma-Aldrich, St. Louis, MO, USA) with 10 μL pyrogallol (4 mM; Sigma-Aldrich, St. Louis, MO, USA) and 130 μL Tris-HCl buffer solution [50 mM, pH 8.2 (Apolo, Taipei, Taiwan), containing 1 mM DTPA (diethylenetriaminepentaacetic acid; Tokyo Chemical Industry Co., Ltd., Tokyo, Japan)], and ddH_2_O. A standard curve was plotted to calculate SOD activities (unit/mg protein). The percentage of SOD inhibition was enumerated as follows:

(9) $$Inhibition\left(\%\right)=\left[1-\left(sample\;or\;standard{∆}_{420\;nm/min}/\max speed\;{∆}_{420\;nm/min}\right)\right]\times 100\%$$

### 3.15. Glutathione Peroxidase (GPx) Assay

The GPx activity was conducted using the RANSEL assay kit (Randox Laboratories Ltd., Crumlin, UK) as referred to by Paglia and Valentine [69]. Cumene hydroperoxide (8 µL) and the reagent (200 µL) were mixed with brain homogenate (4 µL). The absorbance measured that the GPx activity decreased with the initial period (A_0_) and 1.5 (A_1_._5_) min at 340 nm.

### 3.16. Histopathological Sections and Staining

The brain tissue was sectioned and soaked in 10% formalin solution (Merck Millipore Co., Darmstadt, Hesse, Germany) for 24 h before being dehydrated in alcohol (30–99.5%; Sigma-Aldrich) and cleared in xylene (Merck Millipore Co., Darmstadt, Hesse, Germany). Brain tissue was then entrenched in paraffin wax (Leica Microsystems, Singapore) using a digital dry bath incubator (Genepure Technology, Taipei, Taiwan) at 63 °C. The paraffin block was cut into 5 µm thicknesses using a microtome (Model#: HM315R, Thermo Fisher Scientific, Inc., Waltham, MA, USA), transferred to a water bath (43 °C), and dried on a heating plate (35 °C). Slices were dewaxed in xylene for 20 min, rehydrated with graded alcohol, and stained with hematoxylin (Merck Millipore Co., Darmstadt, Hesse, Germany) and eosin (Merck Millipore Co., Darmstadt, Hesse, Germany) for 20 s and 20 min, respectively. After staining, slides were observed and captured under a LEICA DM500 microscope (Leica Microsystems, Singapore) equipped with an IHD-4600 camera system (Sage Vision Co., LTD, New Taipei City, Taiwan) and Toup View 3.7 software (ToupTek Co., LTD, Hangzhou, China).

### 3.17. Statistical Analysis

Data were stated as mean ± standard deviation. A statistical analysis of proximate hydrolysates parameters, antioxidants, ACE-I, AChE, and PEP inhibition was performed with a one-way analysis using the SPSS 22.0 (Statistical Product and Service Solutions) package (SPSS Statistical Software, Inc., Chicago, IL, USA). Tukey’s test determined the significance level at *p* < 0.05.

Authors should discuss the results and how they can be interpreted from the perspective of previous studies and of the working hypotheses. The findings and their implications should be discussed in the broadest context possible. Future research directions may also be highlighted.

## 4. Conclusions

The BIOPEP database revealed that papain was predicted to hydrolyze protein effectively and generate bioactive peptides. Porcine-hydrolyzed with papain (PP) was notable for its superior bioactive properties. Among the tested hydrolysates, PP1 exhibited the highest ACE-I inhibitory activity, making it a prime candidate for the further analysis of its bioactive potential. The administration of PP1 in DG-induced mice significantly improved cognitive performance and memory retention. Additionally, a histological analysis of the hippocampus indicated that PP1 reduced neuronal damage in the dentate gyrus, aligning with the observed cognitive improvements. A further analysis also showed that PP1-treated mice increased SOD and GPx activities, suggesting a reduction in oxidative stress. Overall, this study provides substantial evidence for the use of gelatin hydrolysates, particularly PP1, as promising candidates for developing functional foods or therapeutic agents aimed at treating hypertension, enhancing cognitive function, and protecting against neurodegenerative diseases.

## Figures and Tables

**Table 1:** The frequency (A_E_) of potential antioxidant, angiotensin-converting enzyme (ACE), anti-amnestic, and dipeptidyl peptidase IV inhibitor peptides identified using BIOPEP-UWM’s profiles of potential biological activity tools.

Bioactivities	Bovine		Porcine		Tilapia	
	Bromelain	Papain	Bromelain	Papain	Bromelain	Papain
Antioxidant	0.0014	0.0007	0.0014	0.0007	0.0014	0.0014
ACE inhibitor	0.0971	0.1053	0.0969	0.1091	0.0919	0.1113
Anti-amnestic	0.0410	0.0178	0.0402	0.0177	0.0428	0.0207

A_E_ = d/N, d = number of peptides with specific bioactivity (e.g., antioxidant) released by a given enzyme (e.g., bromelain), and N = number of amino acid residues in a protein.

**Table 2:** Proximate composition (%) of commercial gelatin obtained from bovine, porcine, and tilapia.

Composition	Bovine (B)	Porcine (P)	Tilapia (T)
Moisture	13.29 ± 0.00 ^a^	14.63 ± 0.18 ^c^	14.01 ± 0.07 ^b^
Ash	0.42 ± 0.01 ^c^	0.04 ± 0.01 ^a^	0.21 ± 0.00 ^b^
Crude Protein	83.56 ± 0.60 ^a^	83.31 ± 0.37 ^a^	85.10 ± 0.82 ^b^
Crude fat	2.38 ± 0.24 ^a^	2.50 ± 0.23 ^a^	2.65 ± 0.01 ^a^

Results are presented as mean ± standard deviation (*n* = 3). Different letters (^a,b,c^) within a similar row in each sample group indicate significant differences (*p* < 0.05).

**Table 3:** Characterization gelatin hydrolysates, antioxidant, and ACE-I activities from bovine, porcine, and tilapia digested by bromelain, collagenase, and papain, respectively, and porcine–papain hydrolysates at 1 and 4 h of hydrolysis.

Sample	DH (%)	Protein Contents (%)	Yield *** (%)	DPPH Scavenging Activity (%)	FRAP Activity (mM Fe^2+^/mg Protein)	Metal Ion Chelating Activity (%)	SRSA Activity (%)	ACE-I Activity (%)
Gelatin Hydrolysates *								
Bovine–Bromelain (BB)	24.17 ± 0.03 ^a^	69.49 ± 0.22 ^a^	29.84 ± 0.93 ^a^	19.20 ± 1.77 ^def^	0.073 ± 0.00 ^cde^	13.24 ± 0.40 ^a^	14.89 ± 1.32 ^ab^	27.73 ± 2.06 ^a^
Bovine–Collagenase (BC)	48.95 ± 0.26 ^h^	76.13 ± 0.20 ^b^	59.87 ± 4.31 ^b^	22.53 ± 2.81 ^f^	0.086 ± 0.00 ^ef^	46.66 ± 0.23 ^e^	32.82 ± 1.32 ^e^	44.15 ± 3.75 ^b^
Bovine–Papain (BP)	27.52 ± 0.43 ^b^	75.58 ± 1.86 ^b^	53.49 ± 4.71 ^b^	19.77 ± 0.72 ^ef^	0.090 ± 0.00 ^f^	24.02 ± 0.50 ^b^	20.99 ± 0.00 ^c^	35.62 ± 2.07 ^ab^
Porcine–Bromelain (PB)	43.27 ± 0.97 ^f^	87.17 ± 0.45 ^ef^	59.51 ± 0.92 ^b^	14.60 ± 0.20 ^abc^	0.086 ± 0.01 ^ef^	32.36 ± 0.83 ^c^	16.03 ± 0.66 ^b^	34.08 ± 0.64 ^ab^
Porcine–Collagenase (PC)	53.89 ± 0.53 ^i^	84.35 ± 1.98 ^de^	61.77 ± 9.84 ^b^	18.05 ± 0.72 ^cde^	0.051 ± 0.00 ^ab^	60.95 ± 0.23 ^g^	32.44 ± 1.15 ^e^	44.86 ± 1.08 ^ab^
Porcine–Papain (PP)	37.46 ± 0.24 ^d^	89.57 ± 0.75 ^f^	62.52 ± 2.85 ^b^	15.63 ± 0.40 ^bcd^	0.079 ± 0.00 ^efg^	34.41 ± 1.00 ^d^	21.76 ± 1.75 ^c^	60.94 ± 2.94 ^c^
Tilapia–Bromelain (TB)	31.26 ± 0.53 ^c^	87.54 ± 0.91 ^ef^	67.98 ± 15.67 ^b^	11.38 ± 1.92 ^a^	0.063 ± 0.01 ^bc^	56.25 ± 0.98 ^f^	12.21 ± 1.75 ^a^	38.40 ± 0.53 ^ab^
Tilapia–Collagenase (TC)	45.44 ± 0.32 ^e^	79.02 ± 1.58 ^bc^	60.25 ± 6.92 ^b^	13.56 ± 1.11 ^ab^	0.043 ± 0.00 ^a^	69.76 ± 0.11 ^h^	29.01 ± 0.11 ^d^	48.74 ± 1.14 ^bc^
Tilapia–Papain (TP)	42.30 ± 0.15 ^g^	82.64 ± 1.02 ^cd^	60.30 ± 3.84 ^b^	15.52 ± 0.34 ^bcd^	0.068 ± 0.00 ^cd^	34.22 ± 1.13 ^d^	22.90 ± 0.66 ^c^	39.32 ± 4.69 ^ab^
Porcine–Papain (PP) Hydrolysates **								
PP1 (PP 1-hour hydrolysis)	29.82 ± 0.17 ^a^	94.48 ± 0.55 ^b^	65.23 ± 12.81 ^a^	8.53 ± 0.91 ^a^	0.085 ± 0.01 ^a^	11.83 ± 0.91 ^a^	18.53 ± 0.36 ^a^	96.56 ± 1.37 ^a^
PP4 (PP 4-hour hydrolysis)	37.46 ± 0.24 ^b^	89.11 ± 2.86 ^a^	62.52 ± 2.85 ^a^	8.08 ± 0.46 ^a^	0.068 ± 0.02 ^b^	16.74 ± 1.02 ^a^	17.69 ± 1.31 ^a^	94.76 ± 2.00 ^a^

* DPPH scavenging activity was tested at a concentration of 0.45 mg/mL; FRAP, Metal ion, and SRSA chelating activity were tested at 1.5 mg/mL; ACE-I activity was tested at 2 mg/mL. ** Antioxidant (DPPH, FRAP, metal ion, and SRSA chelating activities) and ACE-I activities were measured at 1 mg/mL. *** Yield: W2 (g)/W1 (g) × 100% (W1: weight of gelatin, W2: weight of hydrolysate after lyophilizing). Results are presented as mean ± standard deviation (*n* = 3). Different letters (^a,b,c,d,e,f,g,h,i^) within a similar row in each sample group (gelatine hydrolysates and PP hydrolysates) indicate significant differences (*p* < 0.05).

**Table 4:** Amino acid composition of the hydrolysate from porcine gelatin hydrolyzed by papain for one hour (PP1).

Amino Acid	PP1 Hydrolysate (g/100 g)
Alanine	7.96 ± 0.09
Arginine	7.20 ± 0.30
Aspartic acid	5.45 ± 0.12
Cystine	0.10 ± 0.00
Glutamic acid	9.48 ± 0.23
Glycine	20.63 ± 0.40
Histidine	0.62 ± 0.03
Isoleucine	1.17 ± 0.00
Leucine	2.55 ± 0.00
Lysine	3.75 ± 0.04
Methionine	0.80 ± 0.03
Phenylalanine	1.72 ± 0.12
Proline	12.23 ± 0.23
Serine	3.44 ± 0.18
Threonine	1.76 ± 0.05
Tryptophan	–
Tyrosine	0.82 ± 0.05
Valine	2.13 ± 0.01

**Table 5:** In vitro analysis on antioxidant, ACE-I, AChE, and PEP inhibitory activities of ultrafiltration fractions (<1 kDa, 1–5 kDa, and 5–10 kDa) from 1 h papain-hydrolyzed porcine gelatin hydrolysates (PP1).

Bioactivities *	PP1 Fractions		
	<1 kDa	1–5 kDa	5–10 kDa
**Antioxidant**			
-DPPH-scavenging activity (%)	ND **	2.95 ± 0.20	ND
-Metal ion chelating activity (%)	ND	3.97 ± 0.58	4.57 ± 0.58
-FRAP activity (mM Fe^2+^/mg protein)	0.22 ± 0.01 ^b^	0.17 ± 0.01 ^a^	0.17 ± 0.01 ^a^
-SRSA activity (%)	18.24 ± 0.44 ^b^	15.47 ± 0.00 ^a^	14.72 ± 1.31 ^a^
**Antihypertensive**			
-ACE-I inhibition	87.42 ± 3.20 ^c^	69.47 ± 4.87 ^b^	10.82 ± 1.96 ^a^
**Anti-amestic**			
-AChE inhibition	21.24 ± 2.36 ^b^	2.30 ± 2.76 ^a^	3.02 ± 1.40 ^a^
-PEP inhibition	48.07 ± 13.65	ND	ND

* Antioxidants, ACE-I inhibitions, and PEP inhibition were determined at 1 mg/mL; AChE inhibition was tested at a concentration of 10 mg/mL. ** Not detectable: negative activity. Results are presented as mean ± standard deviation (*n* = 3). Different letters (^a,b,c^) within the similar row indicate significant differences (*p* < 0.05).

**Table 6:** Treatment of ICR mice groups, physical condition, and organs weight, as well as antioxidant capacities in the brain of the experimental mice.

Parameters	ICR Mice Groups			
	CON	DG	DG_LPP1	DG_HPP1
Initial weight (g)	36.38 ± 0.94 ^a^	36.48 ± 0.71 ^a^	37.08 ± 0.87 ^a^	37.08 ± 0.99 ^a^
Final weight (g)	40.92 ± 2.23 ^a^	38.85 ± 0.95 ^a^	39.41 ± 1.58 ^a^	39.78 ± 1.61 ^a^
Food intake (g/mouse/day)	6.46 ± 0.43 ^a^	6.30 ± 0.33 ^a^	6.41 ± 0.31 ^a^	6.68 ± 0.22 ^a^
Water intake (g/mouse/day)	8.56 ± 0.39 ^a^	8.32 ± 0.37 ^a^	8.72 ± 0.50 ^a^	8.88 ± 0.25 ^a^
Brain (g/100 g BW)	1.33 ± 0.08 ^a^	1.28 ± 0.07 ^a^	1.26 ± 0.07 ^a^	1.29 ± 0.07 ^a^
Liver (g/100 g BW)	3.98 ± 0.14 ^a^	4.14 ± 0.18 ^a^	4.14 ± 0.18 ^a^	4.12 ± 0.05 ^a^
Epididymal fat (g/100 g BW)	0.52 ± 0.12 ^a^	0.46 ± 0.12 ^a^	0.48 ± 0.17 ^a^	0.48 ± 0.17 ^a^
Perirenal fat (g/100 g BW)	1.30 ± 0.22 ^a^	1.00 ± 0.14 ^a^	1.07 ± 0.30 ^a^	1.03 ± 0.15 ^a^
TBARS (nmol MDA eq/mg protein)	31.97 ± 1.67 ^a^	30.68 ± 2.65 ^a^	32.76 ± 1.07 ^a^	31.46 ± 0.84 ^a^
TEAC (nmol Trolox equivalents/mg protein)	300.81 ± 12.07 ^a^	269.59 ± 10.50 ^a^	280.28 ± 15.97 ^a^	277.84 ± 16.58 ^a^
SOD activity (unit/mg protein)	2.03 ± 0.24 ^a^	1.34 ± 0.13 ^b^	1.75 ± 0.08 ^ab^	1.88 ± 0.15 ^a^
GPx activity (munit/mg protein)	49.00 ± 1.61 ^a^	37.58 ± 2.44 ^b^	43.14 ± 2.71 ^ab^	42.66 ± 2.89 ^ab^

Data are given as mean ± standard deviation (*n* = 6). Different letters (^a,b^) within the similar row indicate significant differences (*p* < 0.05).

**Table 7:** Grouping and treatment of ICR mice for the animal behavior assessment.

Group.	Subcutaneous Injection on the Back	Oral Gavage
CON	Saline (0.9%)	Distilled water
DG	D-galactose (300 mg/kg BW/day)	Distilled water
DG_LPP1	D-galactose (300 mg/kg BW/day)	PP1 (100 mg/kg BW/day)
DG_HPP1	D-galactose (300 mg/kg BW/day)	PP1 (500 mg/kg BW/day)

CON: control; DG: D-galactose, DG_LPP1: G-galactose with low concentration of sample, and DG_HPP1: G-galactose with high concentration of sample.

## Data Availability

Data are contained within the article and Supplementary Material.

## Supplementary Materials

The following supporting information can be downloaded at: https://www.mdpi.com/article/10.3390/molecules29184402/s1.
