# Supplementary material for: Bioactive Properties of Enzymatic Gelatin Hydrolysates Based on In Silico, In Vitro, and In Vivo Studies
Source: Molecules. 2024 Sep 16;29(18):4402. doi: 10.3390/molecules29184402 (PMC11434199; doi:10.3390/molecules29184402)
Supplement: Supplementary file 1 [file molecules-29-04402-s001.zip › molecules-3105860-supplementary.pdf]

*Article*

# Bioactive Properties of Enzymatic Gelatin Hydrolysates Based on In Silico, In Vitro, and In Vivo Studies

**Fenny Crista A. Panjaitan [1], Sin-Ting Shie [2], Sung Hoon Park [3], Tesalonika Sevi [2], Wen-Ling Ko [2], Rotimi E. Aluko [4,*] and Yu-Wei Chang [2,*]**

1 Marine Products Processing Study Program, Marine and Fisheries Polytechnic of Jembrana, Bali 82218, Indonesia; fennycap@gmail.com

2 Department of Food Science, National Taiwan Ocean University, Keelung 20224, Taiwan; cindy70255@gmail.com (S.-T.S.); tesalonika.sevi@gmail.com (T.S.); sunnyko0414@gmail.com (W.-L.K.)

3 Department of Food and Nutrition, College of Life Sciences, Gangneug-Wonju National University, 25457, Republic of Korea; sungpark@gwnu.ac.kr

4 Department of Food and Human Nutritional Sciences, University of Manitoba, Winnipeg, MB R3T2N2, Canada

* Correspondence: rotimi.aluko@umanitoba.ca (R.E.A.); bweichang@mail.ntou.edu.tw (Y.-W.C.); Tel.: +1-204-290-2963 (R.E.A.); +886-2-2462-2192 ext. 5152 (Y.-W.C.)

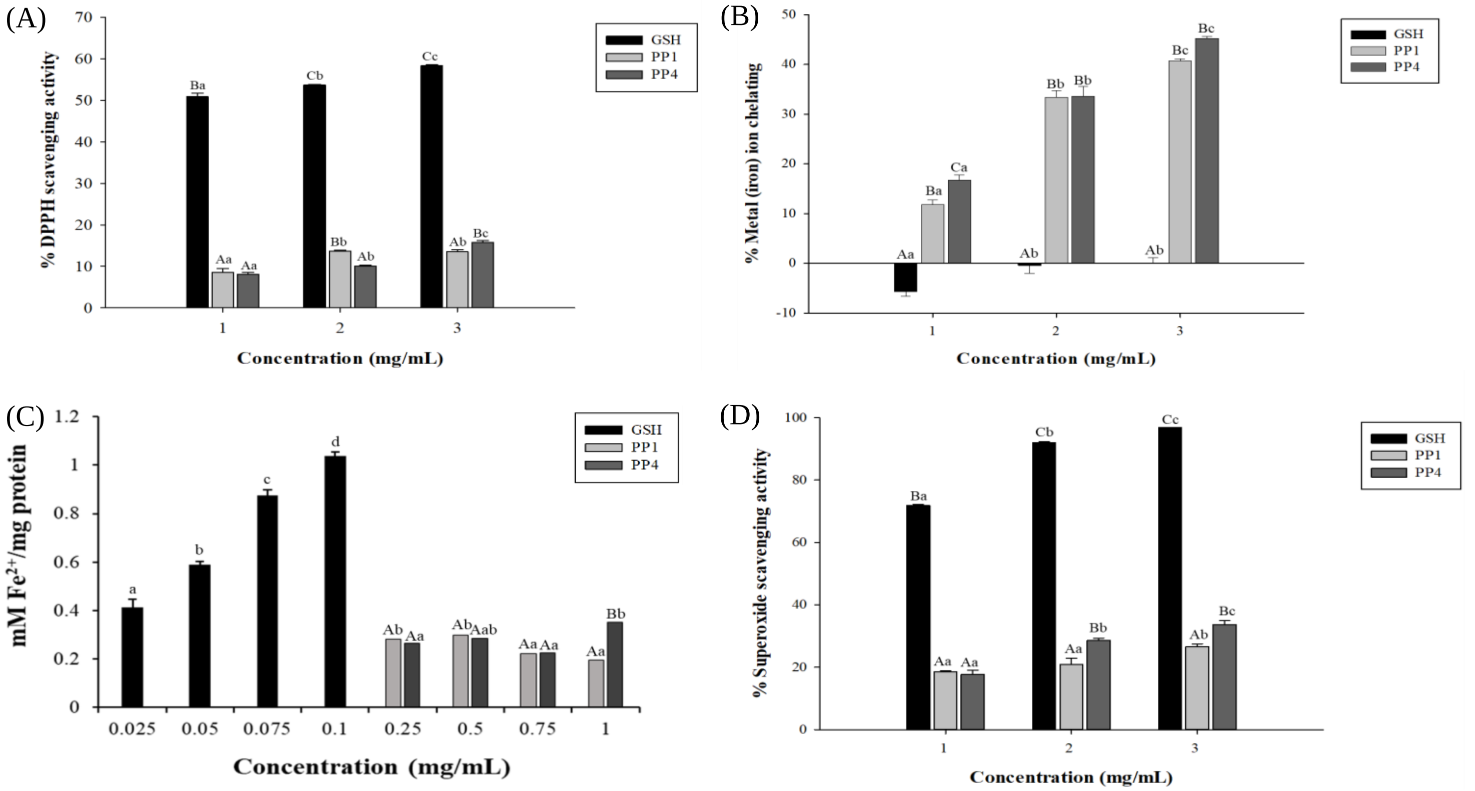


**Figure S1.** *In vitro* antioxidant activities, (A) DPPH radical scavenging activity, (B) Metal ion chelating, (C) Ferric reducing antioxidant power, (D) Superoxide radical scavenging activity of porcine gelatin hydrolysates generated by papain at 1 hr and 4 hr. GSH used as the control. Bars with different letters are significantly different in the same protein concentration (capital letters; A,B,C) or sample (small letters; a,b,c,d) at $p < 0.05$..
